# Hantavirus Infections in Humans and Animals, China

**DOI:** 10.3201/eid1608.090470

**Published:** 2010-08

**Authors:** Yong-Zhen Zhang, Yang Zou, Zhen F. Fu, Alexander Plyusnin

**Affiliations:** State Key Laboratory for Infectious Disease Control and Prevention, Beijing, People’s Republic of China (Y.-Z. Zhang, Y. Zou); University of Georgia, Athens, Georgia, USA (Z.F. Fu); University of Helsinki, Helsinki, Finland (A. Plyusnin)

**Keywords:** China, Hantavirus, Bunyaviridae, HFRS, natural hosts, viruses, zoonoses, perspective

## Abstract

Hemorrhagic fever with renal syndrome (HFRS) is a serious public health problem in the People’s Republic of China. Although 7 sero/genotypes of hantaviruses have been found in rodents, only Hantaan virus (carried by *Apodemus agrarius* mice) and Seoul virus (carried by *Rattus norvegicus* rats) reportedly cause disease in humans. During 1950–2007, a total of 1,557,622 cases of HFRS in humans and 46,427 deaths (3%) were reported in China. HFRS has been reported in 29 of 31 provinces in China. After implementation of comprehensive preventive measures, including vaccination, in the past decade in China, incidence of HFRS has dramatically decreased; only 11,248 HFRS cases were reported in 2007. Mortality rates also declined from the highest level of 14.2% in 1969 to ≈1% during 1995–2007. However, the numbers of HFRS cases and deaths in China remain the highest in the world.

## Abstract 2

Hemorrhagic fever with renal syndrome is a serious public health problem in China.

During the past decade, hantaviruses have gained worldwide attention as emerging zoonotic pathogens (*1**–**3*). Hantaviruses, which belong to the family *Bunyaviridae*, genus *Hantavirus*, are enveloped, single-stranded, negative-sense RNA viruses. Transmission among rodents and from rodents to humans generally occurs through inhalation of aerosolized excreta (*4*). In their natural hosts (rodents of the families *Muridae and Cricetidae), h*antaviruses establish a persistent infection, which causes no apparent harm (*5*). In humans, however, hantaviruses cause 2 diseases: hemorrhagic fever with renal syndrome (HFRS) in Eurasia, and hantavirus (cardio)pulmonary syndrome in North and South America (*6*). Each year worldwide, 60,000–100,000 HFRS cases are reported, mostly from the People’s Republic of China (*7*).

To date, 7 sero/genotypes of hantaviruses have been identified in China (*8*). Of these, only Hantaan virus (HTNV), carried by *Apodemus agrarius* mice, and Seoul virus (SEOV), carried by *Rattus norvegicus* rats, cause HFRS (*8**–**11*). Despite intensive measures implemented in the past 3 decades, HFRS remains a major public health problem in China (*10*).

## Incidence and Mortality Rates

**Figure 1:**
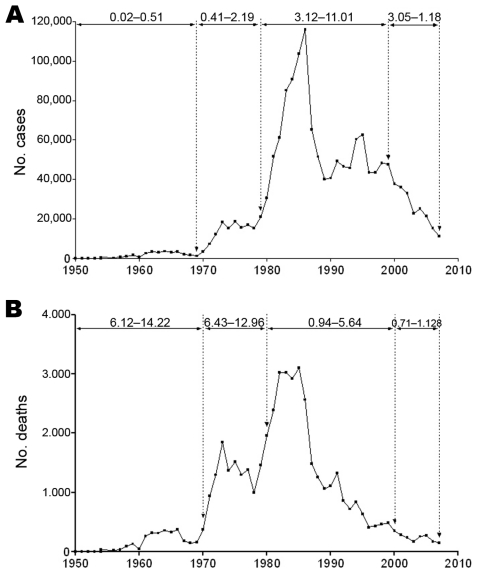
Annual numbers of hemorrhagic fever with renal syndrome (HFRS) cases (A) and HFRS-caused deaths (B) reported in China, 1950–2007. Incidence rates are cases/100,000 population. Mortality rates are shown at top.

HFRS-like disease was described in Chinese writings ≈1,000 years ago. Then in the early 1930s, HFRS cases among Japanese soldiers in northeastern China were reported (*9*). Subsequently, HFRS cases have been reported each year in China, >30,000 cases during 1931–1949 (*9*). Since 1950, HFRS has been listed as a class B notifiable disease. Before 1982, HFRS cases were defined by a national standard of clinical criteria; and starting in 1982, cases were also confirmed by detection of antibodies against hantavirus in patients’ serum samples. Serious epidemics occurred during the 1980s and 1990s (*9**,**10*). During the 58-year period of 1950–2007, a total of 1,557,622 HFRS cases were reported in China (Figure 1, panel A). Only a few cases were reported in the beginning of the 1950s, after which the number gradually increased. The first peak was reported in 1964 (3,520 cases; 0.5 cases/100,000 population), and then the number declined gradually to only 1,139 cases (0.1 cases/100,000 population) in 1969. HFRS cases again increased in the beginning of the 1970s. During 1970–1979, a total of 143,949 cases were reported, representing a >6-fold increase over the number reported in the 1960s (23,824). The actual number of HFRS cases might be even higher for these periods because the reporting system was suboptimal and knowledge of pathogen source, transmission routes, and diagnostics was poor. The third peak was reported in 1986, when 115,804 cases were reported (11.1 cases/100,000 population), the largest annual number of HFRS cases during the 58-year period. During 1980–1989, a total of 696,074 cases were reported. During the 1990s, the total number of cases was reduced to 488,135 (29.9% reduction from cases in 1980–1989); the annual number of cases fluctuated between 40,000 and 62,754. The fourth peak was reported during 1994–1995, at >60,000 cases/year. Since 2000, the annual number of HFRS cases has declined >3-fold, from 37,814 in 2000 to 11,248 in 2007.

The number of deaths from HFRS during these same 58 years, 1950–2007, totaled 46,427 (Figure 1, panel B); median death rate was 3%. Death rates varied substantially, from 14.2% in 1969 to 5.6% in 1981. The high death rates reflected not only the severity of HFRS caused by HTNV but also the poor knowledge of how to treat it. Death rates declined gradually, from 4.9% in 1982 to 2.7% in 1991, then to 1% in 1995, and have remained at ≈1% during the past decade (1996–2007). The accumulated knowledge about HFRS and improved diagnostics and treatment have dramatically increased survival rates. In addition, the gradual change in the disease structure (proportions of mild and severe disease) might have contributed to the decreased mortality rates as well. In recent decades, as rats (*Rattus* spp.) followed human activities and migration from rural to urban areas during the fast socioeconomic development in China, the proportion of mild HFRS cases caused by SEOV steadily increased while the proportion of more severe cases associated with HTNV infection decreased (*9**,**10**,**12**–**14*). Increased awareness of diagnosis, treatment, and prevention also contributed to the decrease of the more severe cases.

## Geographic Distribution

**Figure 2:**
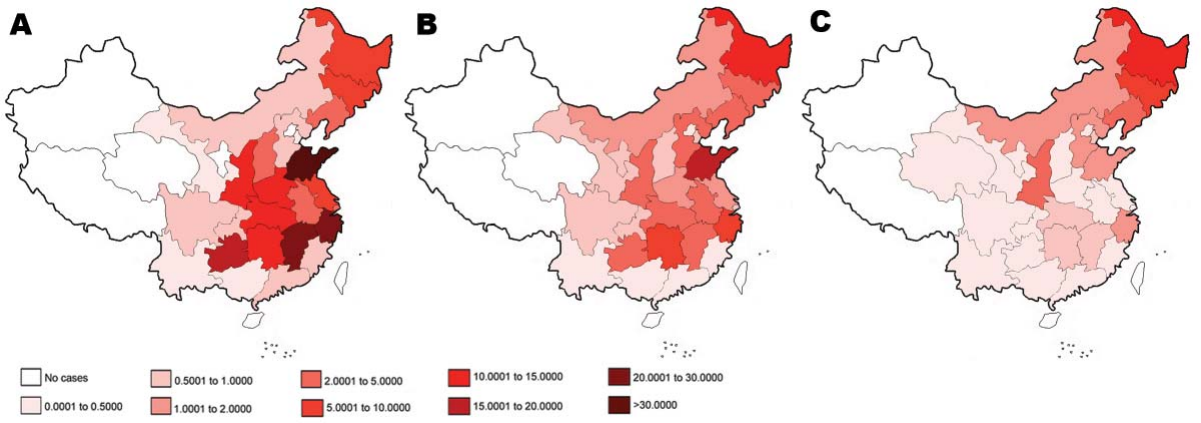
Geographic distribution and annual incidence of hemorrhagic fever with renal syndrome in China in 1986 (A), 1996 (B), and 2006 (C).

Before 1950, HFRS cases had been reported in only 2 provinces (*9*), Heilongjiang and Jilin, which are located in northeastern China and share borders with Russia and North Korea, respectively. By the end of the 1950s, sporadic HFRS cases were reported in 8 provinces, spreading southward from northeastern to eastern and central China. By the end of the 1960s, HFRS cases were noted in 18 provinces; by the end of the 1970s, in 19 provinces; and during the 1980s and 1990s, in 27 and 28 provinces in southern and southwestern China, respectively. Especially after the discovery of SEOV in 1981 (*15*), HFRS distribution became nationwide; only 3 provinces (Qinghai, Xizang, and Xinjiang) remained unaffected (Figure 2). During 2000–2007, HFRS cases declined dramatically (Figure 2, panel C). Since 2005, however, 2 HFRS cases have been reported in Qinghai Province, where HFRS had never been found before; further studies are needed to clarify whether these 2 cases were indigenous or imported.

Although HFRS cases have been found in 29 provinces, the disease remains more prevalent in Shandong, Heilongjiang, Jilin, Liaoning, Hebei, Jiangsu, Zhejiang, Anhui, Henan, Jiangxi, Hubei, Hunan, Shaanxi, Sichun, and Guizhou provinces (Figure 2). Cases in these 15 provinces account for ≈95% of all HFRS cases reported since 1950, and each province reported >1,000 cases during 1990–1999. Most HFRS cases occurred in Shandong Province, which had 23.7%, 7.9%, 36.1%, and 27% of cases in China in 1985, 1995, 1990 and 2000, respectively. Since implementation of comprehensive preventive measures in 1981, incidence of HFRS has substantially decreased in Anhui, Guizhou, Henan, Hubei, Hunan, Jiangsu, Jiangxi, Shangdong, and Sichuan provinces in the past 8 years but remains high in Heilongjiang, Jilin, Liaoning, Hebei, Shaanxi, Shangdong, Inner Mongolia, and Zhejaing provinces (Figure 2, panel C).

Although during the past decade, the annual incidence of HFRS has been gradually decreasing in China, the disease has emerged in areas where it had not been reported during the periods of high prevalence (1980–1990s), such as the Bayannaoer District of Inner Mongolia (*14*). In addition, incidence of HFRS caused by SEOV has been high in some cities, e.g., Beijing and Shenyang (*10**,**16**,**17*).

## Epidemiology

### Infections in Humans

For humans, individual HFRS cases as well as outbreaks are influenced by natural (e.g., ecologic) and occupational factors (*3**,**18**,**19*). Many hantavirus infections occur in persons of low socioeconomic status because of poor housing conditions (*6*). In China as well, occurrence and epidemics of HFRS are influenced by natural and social factors (*10**,**14**,**20**–**22*). HFRS cases occur mainly in the northeastern, eastern, central, and southwestern parts of China (humid and semihumid zones) and rarely in the northwestern part (arid zone) (Figure 2). Rural areas account for >70% of HFRS cases; i.e., mainly peasants are infected (*9**,**10*). Poor housing conditions and high rodent density in residential areas seem to be responsible for most HFRS epidemics.

The increase of HFRS from the end of the 1970s coincided with the fast socioeconomic development that started in 1978 in China. During the 1980s and 1990s, China underwent large changes such as agricultural development, irrigation engineering, urban construction, mining, and highway and railway construction. These activities might increase human contact with rodents. Because rats are more mobile than other hantavirus hosts (*4*), fast socioeconomic development also causes wide dispersion of rats and SEOV (*23*), which might subsequently lead to the high nationwide prevalence of SEOV infections. However, improved housing conditions, improved hygiene, and human migration from rural areas to cities might contribute the declining trend of HFRS cases since 2000.

Generally, HFRS cases occur throughout the year and increase in winter and spring (*9**,**10**,**12*). Early epidemiologic investigations found that the winter peak resulted from HTNV carried by striped field mice and that the larger spring epidemic was mainly caused by SEOV carried by Norway rats (*12*).

The age of HFRS patients is all inclusive (from infancy to >65 years), but mostly adolescents and young adults are affected (*9**,**10**,**12*). During 1997–2003, among 265,691 HFRS cases reported and confirmed by epidemiologic surveys, 4.2% were in children <14 years of age, 91.2% were in persons 15–64 years of age, ≈4.5% were in persons >65 years of age, and 0.1% were of persons of unknown age. Of these patients, 70.63% were male.

In addition to hantavirus transmission to humans from wild rodents, HFRS outbreaks associated with laboratory animals have been reported in China (*17**,**24*). In 1983, laboratory rats accounted for hantavirus transmission, resulting in 16 HFRS cases in 1983 in Shanxi Province (*25*). Since then, dozens of hantavirus infections associated with laboratory rodents have occurred (*17*) and increased during recent years (*26*). For example, in 2006 an outbreak of HFRS among students in Shenyang was caused by SEOV that had been circulating in local wild rats (*R. norvegicus*) and was transmitted to humans through laboratory rats (*17*). Because SEOV is prevalent in urban areas of China, surveillance of hantavirus infection in laboratory rodents and management of laboratory animal centers should be reinforced to prevent laboratory-associated cases of HFRS.

### Infections in Animals

Species diversity of rodents and insectivores in China is remarkable (*27*). A total of 171 species of rodents, which belong to 10 subfamilies, have been found; the subfamilies Murinae and Microtinae contain 38 and 43 species, respectively. In addition, at least 32 species of insectivores are present. These rodent and insectivore species are distributed nationwide. In particularly, *A. agrarius* and *R. norvegicus* rodents, the reservoir hosts of HTNV and SEOV, are the predominant species (Table 1) and have been found in 28 and 30 provinces, respectively, in China.

**Table 1:** Major rodent species captured in China, by location and years*

Species	Fields, %				Residential areas, %		
	1984–1990	1991–1995	1996–2000		1984–1990	1991–1995	1996–2000
*Apodemus agrarius*	57.362	44.508	37.012		3.425	4.411	3.944
*Apodemus peninsulae*	3.761	8.870	1.516		0.011	0.137	0.125
*Apodemus chevrieri*	0.318	2.475	4.053		NC	1.820	2.282
*Rattus norvegicus*	5.081	8.358	10.112		49.106	53.025	54.266
*Rattus losea*	7.302	6.401	22.457		0.141	0.289	0.087
*Rattus flavipectus*	1.089	2.674	2.284		13.527	10.969	9.395
*Rattus nitidus*	0.225	0.158	0.042		0.006	0.007	0.067
*Niviventer confucianus*	0.775	0.339	0.987		0.138	0.078	0.062
*Niviventer fulvescens*	1.062	0.164	0.184		0.001	NC	NC
*Mus musculus*	4.635	5.889	4.472		28.625	21.045	20.984
*Cricetulus barabensis*	3.883	3.946	1.781		0.427	0.171	0.067
*Cricetulus longicaudatus*	1.124	0.769	NC		0.033	NC	NC
*Cricetulus migratorius*	0.784	1.097	1.120		0.342	0.022	0.766
*Cricetulus triton*	7.532	8.065	6.282		1.458	3.911	2.657
*Meriones meridianus*	0.513	NC	NC		NC	NC	NC
*Meriones erythrourus*	0.445	0.070	0.884		0.053	NC	0.004
*Myodes rutilus*	0.230	0.091	0.558		0.030	0.022	0.017
*Myodes rufocanus*	0.185	0.319	0.194		0.035	0.037	0.092
*Microtus fortis*	0.469	1.340	1.368		0.005	NC	NC
*Microtus maximowiczii*	0.274	0.219	0.174		0.073	0.056	0.046
*Microtus arvalis*	0.176	0.155	0.052		NC	NC	0.208
*Anourosorex squamipes*	0.429	0.342	NC		0.005	2.024	3.919
*Crocidrua lasiura*	0.418	0.202	0.161		0.012	0.041	0.062
*Crocidrua horsfieldi*	0.006	0.544	0.026		NC	0.204	0.042
*Suncus murinus*	0.354	0.427	1.520		1.826	0.489	0.183
*Sorex araneus*	0.219	0.187	0.303		0.044	0.037	0.258

*Data from Chen and Luo (*23*). Hantavirus antigens had been identified in all species listed except *Anourosorex squamipes* mole shrews and *Microtus arvalis* voles. NC, not captured.

National HFRS surveillance data (1984–2000) and the nationwide geographic epidemiologic investigation of HFRS (1984–1987) have detected hantavirus antibodies or antigens in 67 species of vertebrates (*23**,**28*). Of those, 38 species of rodents and 8 species of insectivores had been found to contain hantavirus antigen. Hantavirus infection has been reported for several species of domestic animals (e.g., cats, pigs, rabbits, dogs) as well. Most recently, we found hantavirus antigen in lung tissue of midday jirds (*Meriones meridianus*), which belong to family Muridae, subfamily Gerbilinae, which have not been known to carry hantaviruses (*14*). Thus, yet-unidentified hantaviruses may be circulating in China.

During 1984–2000, a total of 167,540 small animals were trapped in the wild (mostly *A. agrarius* mice) and 184,096 rodents were trapped in residential areas (mostly *R. norvegicus* rats), as recorded by national surveillance centers (*23*) (Table 2). Of the small animals, 10,238 had hantavirus antigen. *A. agrarius* mice accounted for 44.9% (3,270/7,282) of hantavirus antigen–positive animals collected during 1984–1990, 59.55% (970/1,629) of those collected during 1991–1995, and 32% (424/1,327) of those collected during 1996–2000. *R. norvegicus* rats accounted for 37.2% (2,707/7,282), 23.9% (390/1,629), and 43 (570/1,327) of hantavirus antigen–positive animals during corresponding periods. Hantavirus-positive *A. agrarius* mice have been found in all parts of China except Xinjiang Province, and hantivirus-positive *R. norvegicus* rats have been found in most provinces except Qinghai, Xinjiang, and Xizang.

**Table 2:** Hantaviruses circulating in China, 1981–2008

Virus, rodent	Human disease*	Distribution within China
Hantaan		
*Apodemus agrarius* mouse	HFRS (severe)	Nationwide except Xinjiang and Jilin provinces
*A. peninsulae* mouse	HFRS (severe)	Nationwide except Xinjiang and Jilin provinces
Seoul		
*Rattus norvegicus* rat	HFRS (mild)	Nationwide except Qinhai, Xinjiang, Xizang provinces
*R. rattus* rat	HFRS (mild)	Eastern and southwestern parts
*R. flavipectus* rat	HFRS (mild)	Eastern, central, southern, and southwestern parts
*R. losea* rat	HFRS (mild)	Southern and southwestern parts
*R. nitidus* rat	HFRS (mild)	Eastern, central, southern, and southwestern parts
Da Bie Shan, *Niviventer confucianus* rat	Unknown	Anhui Province
Hokkaido, *Myodes rufocanus* vole	Unknown	Jilin Province
Khabarovsk, *Microtus maximowiczii* vole	Unknown	Inner Mongolia
Vladivostok, *Microtus fortis* vole	Unknown	Jilin and Liaoning provinces
Yuanjiang, *M. fortis* vole	Unknown	Hunan Province

*HFRS, hemorrhagic fever with renal syndrome.

## Hantavirus Isolates

**Figure 3:**
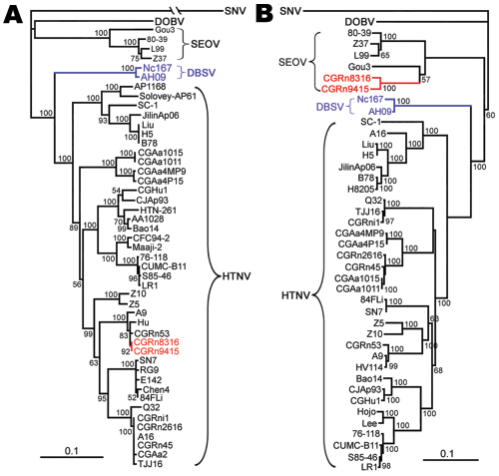
Phylogenetic trees of Hantaan virus (HTNV) variants according to the small segment (A) and medium segment (B) coding sequences. PHYLIP program package version 3.65 (http://helix.nih.gov/Applications/phylip.html) was used to construct the phylogenetic trees; the neighbor-joining method was used. Bootstrap values were calculated from 1,000 replicates; only values >50% are shown at the branch nodes. The trees constructed using the maximum-likelihood method (not shown) had similar topology. Scale bars indicate nucleotide substitutions per site. Colors (blue and red) highlight viruses of interest from China. SNV, Sin Nombre virus; DOBV, Dobrava-Belgrade virus; SEOV, Seoul virus; DBSV, Da Bie Shan virus.

Antigenic and genetic studies of hantaviruses isolated from HFRS patients and rodents in China found 3 hantaviruses in China: HTNV, SEOV, and Da Bie Shan virus carried by Chinese white-bellied rats (*Niviventer confucianus*) (Table 2, Figure 3) (*11**–**13*). Recently, we found Puumala virus-like Hokkaido virus in *Myodes rufocanus* voles (*29*), Khabarovsk virus in *Microtus maximowiczii* voles, Vladivostok virus in *Microtus fortis*, subspecies *pelliceus* voles (*30*), and a presumably novel Yuanjiang virus in *M. fortis,* subspecies *calamorum* voles (*8*). So far, only HTNV and SEOV are known to cause HFRS in China (*8*–*11*). Because *A. agrarius* and *R. norvegicus* rodents are the predominant carriers and are distributed nationwide, HTNV and SEOV are obviously the major threat for HFRS in China.

HTNV was first isolated from striped field mice in 1981 (*31*). Consistent with the geographic distribution of *A. agrarius* mice, HTNV has been found in all Chinese provinces except Xinjiang (*11**,**12**,**22**,**28**,**32*). In addition to *A. agrarius* mice, HTNV has been also found in *Apodemus peninsulae* mice in northeastern China (*33*). Genetic analysis of the small (S) and medium (M) genome segments suggested that at least 9 distinct lineages of HTNV are circulating in China (Figure 3) (*11**,**32*). Generally, HTNV variants display geographic clustering. Recently, we detected reassortment between HTNV and SEOV in *R. norvegicus* rats in Guizhou Province (Figure 3) (*34*), which indicates that genetic reassortment occurs naturally between 2 hantavirus types. Because reassortment is a way for segmented viruses to achieve high infectivity and adapt to new animal hosts, further studies are warranted to evaluate susceptibility of *A. agrarius* and *R. norvegicus* rodents to these unique reassortant viruses and to determine whether these reassortants can infect humans.

**Figure 4:**
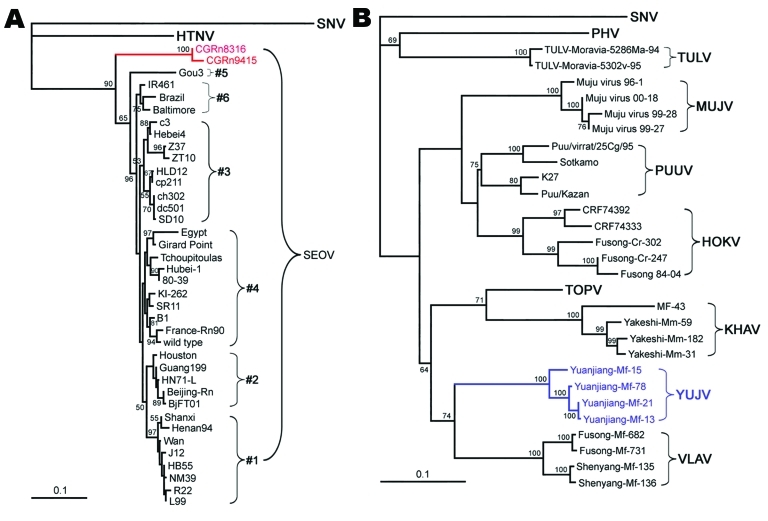
Phylogenetic tree of Seoul virus (SEOV) variants according to partial (nt 2001–2301) medium segment sequences (A). Phylogenetic tree of hantaviruses according to complete coding sequences of the medium segment (B). PHYLIP program package version 3.65 (http://helix.nih.gov/Applications/phylip.html) was used to construct the phylogenetic trees; the neighbor-joining method was used. Bootstrap values were calculated from 1,000 replicates; only values >50% are shown at the branch nodes. The trees constructed by using the maximum-likelihood method (not shown) had similar topology. Scale bars indicate nucleotide substitutions per site. Colors (blue and red) highlight viruses of interest from China. SNV, Sin Nombre virus; HTNV, Hantaan virus; PHV, Prospect Hill virus; SEOV, Seoul virus; TULV, Tula virus; MUJV, Muju virus; PUUV, Puumala virus; HOKV, Hokkaido virus; KHAV, Khabarovsk virus; YUJV, Yuanjiang virus; VLAV, Vladivostok virus.

HFRS cases caused by SEOV were first reported in Henan and Shanxi provinces along the Yellow River in China (*15*). Subsequently, SEOV (strain R22) was isolated from *R. norvegicus* rats in Henan (*35*), and SEOV has been found in almost all provinces of China except Qinghai, Xinjiang, and Xizang (*11**,**14**,**23**,**28*). SEOV-associated HFRS seems to have recently spread to areas where it had not been reported during previous epidemics (*14*). Most known SEOV variants (from lineages 1–4 and 6), including those from China, Brazil, Japan, South Korea, North America, and the United Kingdom, are genetically homogeneous (Figure 4, panel A). Lineages 1–4 are widely distributed and do not follow a geographic clustering pattern. Thus, the variants from lineages 1–4 and 6 are closely related and may share a more recent common ancestor. Because *R. norvegicus* rats are distributed nationwide (*27**,**28*) and are more mobile than other hantavirus hosts (*4*), SEOV has become the largest threat for public heath in China and may bring even more potential threats to humans as rat species become more widespread along with globalization of the economy. Natural HFRS cases caused by SEOV have been found almost exclusively in China and other Asian countries. Lack of HFRS in other countries may result from better living conditions, low rat densities, and low SEOV-carrying rates by the rats.

In 2000, Da Bie Shan virus was isolated from Chinese white-bellied rats captured in the Da Bie San mountainous area of Anhui Province (*11*). Although white-bellied rats are widely distributed in China (*27*), Da Bie Shan virus has not yet been found outside this area. Serologic and genetic analyses have shown it to be distinct from HTNV and other known hantaviruses (*11*). According to current taxonomy, Da Bie Shan virus is a provisional, novel hantavirus species. Whether this virus can be transmitted to humans and cause HFRS remains unknown.

Previous investigations found hantavirus antigens in *M. rufocanus, M. fortis, M. maximowiczii*, and other voles (*9**,**12**,**23**,**28*). Recently, we recovered the S and M segment sequences from *M. rufocanus* voles trapped in Fusong, Jilin Province (Figure 4, panel B) (*29*). Phylogenetic analysis of these sequences revealed that they belong to Hokkaido virus, which was first identified in *M. rufocanus* voles in Hokkaido, Japan (*36*), and form a distinct lineage. Whether Hokkaido virus and other Puumala-like viruses (e.g., Muju virus) are pathogenic in humans is not known, but the possibility cannot be excluded because Puumala virus carried by bank voles (*M. glareolus*) in Europe causes a milder form of HFRS, nephropathia epidemica.

Our recent study found Khabarovsk and Vladivostok viruses in China (Figure 4, panel B) (*30*). The virus isolated from reed voles in Fusong County (Jilin Province) is closely related to Vladivostok virus, whereas the virus isolated from the Maximowiczi vole in Yakeshi (Inner Mongolia) is closely related to Khabarovsk virus. These results suggest that *M. fortis* voles are the natural host for Vladivostok virus and that *M. maximowiczii* voles are the natural host for Khabarovsk virus.

Further molecular investigation showed that hantaviruses detected in *M. fortis* (subsp. *dolichocephalus*) voles from Shenyang belong to Vladivostok virus and form a distinct lineage on the phylogenetic trees on the basis of the S and M segment sequences (Figure 4, panel B) (*8*). Complete S segment and partial large (L) segment sequences from the virus identified in *M. fortis* (subsp. *calamorum*) voles from Yuanjiang (Hunan Province) were distinct from those of Shenyang and Fusong variants; they had up to 18% nucleotide and 5% amino acid sequence divergences. Moreover, partial M segment sequences (nt 2676–3650) from the Yuanjiang variant were even more divergent from Shenyang and Fusong variants (>20% and 8%, respectively). Thus, our results suggest that the hantavirus from *M. fortis* *calamorum* voles in Yuanjiang represents a novel hantavirus species, Yuanjiang virus. These data also demonstrate impressive genetic diversity and complexity of the *M. fortis* vole–associated hantaviruses in China.

Hantaviruses are thought to have coevolved with their respective hosts. Each serotype and/or genotype of hantavirus appears to be primarily associated with 1 (or a few closely related) specific rodent host species (*4*). As described above, >100 species of rodents and several dozens of insectivores are widely distributed in HFRS-endemic areas in China (*27*). Hantavirus-specific antibodies and/or antigens have been identified in at least 38 rodent species (*23**,**28*). Therefore, in addition to already known HTNV, SEOV, Da Bie Shan virus, Hokkaido virus, Khabarovsk virus, Vladivostok virus, and Yuanjiang virus, yet-unknown hantavirus species may be circulating in China. In-depth studies on hantavirus distribution through different geographic regions and hosts in China as well as genetic characterization of hantaviruses and elucidation of the relationship among them and between these viruses and other known hantaviruses should help prevent and control the diseases they cause.

## Control and Prevention

To control and prevent HFRS in China, a comprehensive preventive strategy has been implemented and includes public health education and promotion, rodent control, surveillance, and vaccination (*10*). Surveillance of hantavirus infection in rodents could help with organization of an advanced warning service for possible increases in human infections. In 1984, a national surveillance system was established on mainland China (*12*). Each province to which HFRS is endemic has at least 1 surveillance laboratory; the number depends on the severity of HFRS. According to a request from the Chinese Center for Disease Control and Prevention (previously Chinese Academy of Preventive Medicine), studies have been conducted to determine 1) the number of HFRS cases, 2) the list of local small animal species (including their density in nature and in residential areas), and 3) hantavirus prevalence in rodent and human populations. The system has provided systemic epidemiologic knowledge of hantavirus infection in China.

Inactivated hantavirus vaccine was developed after HTNV and SEOV were successfully isolated and propagated in A-549 cells (*31**,**37**,**38*). Inactivated hantavirus vaccine was first approved in 1993 and, since 1995, has been used in areas where HFRS is highly endemic. Four hantavirus vaccines based on inactivated HTNV and SEOV have been widely used and demonstrated to be safe and efficacious (Table 3) (*39*). Every year, ≈2 million vaccine doses are used. Purified bivalent vaccine for HTNV and SEOV cultured in Vero cells has been used since 2003 (*40*). From 2008, hantavirus vaccine has been included in the national Expanded Program on Immunization. For persons in areas in which HFRS is highly endemic, the vaccination is free of charge.

**Table 3:** Inactivated hantavirus vaccines used in China, 1995–2010

Virus	Cell culture used	Vaccination procedure*	Protection, %
Hantaan	Mongolian gerbil kidney	3 + 1	>90
Seoul	Golden hamster kidney	2 + 1	>95
Hantaan	Suckling mouse brain	3 + 1	>90
Hantaan/Seoul	Mongolian gerbil kidney	2 + 1	>95
Hantaan/Seoul	Vero	2 + 1	>85†

*No. doses + no. boosters. †Seroconversion rate of human hantavirus antibody determined by plaque-reduction neutralization test.

The most effective way to control hantavirus diseases is to reduce human exposure to infected rodents and their excrements. Since the 1950s on mainland China, the rat population has been controlled by using poison bait or trapping around residential areas. During the 1980s and 1990s, deratization around residential areas effectively decreased both rodent density and incidence of HFRS, especially the disease caused by SEOV (*23**,**28*). In addition, the minimization of food availability for rodents around residential areas effectively reduced rodent populations.

Improving general awareness and knowledge of pathogen source, transmission routes (how to avoid contact with a pathogen), diagnostics, vaccination, and general hygiene is one of the most effective and economic ways to prevent infectious diseases. Since the 1970s, public education on HFRS and other infectious diseases has been conducted by all possible means in China, especially in rural areas.

## Conclusions

At least 7 geno/serotypes of hantaviruses are circulating in rodents in China, and, as better tests are developed, more not-yet identified hantaviruses may be found in rodent or insectivore species. Therefore, a better understanding of hantavirus infection ecology and epidemiology would be beneficial for controlling the disease in humans.

Environmental and social economic changes may affect the geographic distribution, abundance, and dynamics of rodent carriers and, hence, the epidemiology of hantavirus infections. Over the past few decades, recognition and understanding of hantavirus infection in China have greatly improved. Although HFRS was highly epidemic during the 1980s and 1990s, the incidence has dramatically declined during the past 8 years as a result of comprehensive preventive measures and improved living conditions. HFRS-associated mortality rates also decreased dramatically. However, the total number of HFRS cases and the number of deaths are the highest in the world, and China still has a long way to go to control hantavirus infection in humans.
